# Novel equations for estimating gross energy in feed ingredients for non-ruminants

**DOI:** 10.5713/ab.24.0312

**Published:** 2024-08-26

**Authors:** Yoon Soo Song, Ah Reum Son, Beob Gyun Kim

**Affiliations:** 1 Department of Animal Science, Konkuk University, Seoul 05029, Korea

**Keywords:** Equation, Feed Ingredient, Gross Energy, Validation

## Abstract

**Objective:**

The present study aimed to evaluate the accuracy of previous equations for estimating gross energy (GE) in feed ingredients and to develop the novel equations.

**Methods:**

A total of 2,279 ingredient samples consisted of barley (n = 58), corn (n = 319), corn distillers dried grains with solubles (n = 13), corn gluten feed (n = 583), copra expellers (n = 156), copra meal (n = 234), cottonseed meal (n = 12), palm kernel expellers (n = 504), rapeseed meal (n = 114), soybean meal (n = 138), wheat (n = 70), and wheat bran (n = 78) were analyzed for dry matter (DM), crude protein (CP), ether extract (EE), crude fiber, ash, and GE. The 2,279 ingredient samples were used for evaluating the previous equations and developing novel equations. Using data from 62 ingredients in the swine NRC publication in 2012, the old equations and the novel equations were evaluated.

**Results:**

Based on the evaluation using 2,279 samples, the equation developed by Ewan in 1989 underestimates GE by 218 kcal/kg DM (standard error = 4 and p<0.001) on average and underestimates more for low-GE ingredients (linear bias = −0.121; standard error = 0.025 and p<0.001). The equation reported by Sauvant, Perez, and Tran in 2004 also underestimates GE by 135 kcal/kg DM (standard error = 4 and p<0.001) on average. Novel equations for estimating GE concentration (kcal/kg DM) in feeds were developed: GE = 4,299+7×CP +53×EE, with R^2^ = 0.342 and p<0.001; GE = 4,341+11×CP+54×EE–24×ash, with R^2^ = 0.372 and p<0.001, where all independent variables are in % DM. In the validation using 62 feed ingredients in the NRC publication, the equations developed in the present study were accurate whereas the previous equations were not.

**Conclusion:**

The novel equations developed in the present study fairly accurately estimate gross energy concentrations in concentrate feeds.

## INTRODUCTION

Energy is the most important component in swine diets comprising the largest proportion of diets and playing a vital role in the maintenance and growth of pigs [1,2]. Digestible (DE), metabolizable (ME), and net energy (NE), which reflect the energy utilizations in pigs, are used to formulate diets that fulfil energy requirements for optimal growth [3]. The DE, ME, and NE values can be estimated using prediction equations that take into account the chemical composition in feeds [1,4–8]. Notably, the accuracy of the most prediction equations improves when the gross energy (GE) concentration is used as an independent variable. Consequently, accurate determination of GE in feeds is essential for effectively estimating DE, ME, and NE for diet formulations.

The GE in feed ingredients can be determined using a bomb calorimeter. This method is relatively inexpensive and accurate, but time-consuming and not commonly conducted in commercial practice [9]. Alternatively, prediction equations for estimating GE in feeds based on their chemical compositions have been developed in previous studies [10,11] and introduced in the swine NRC [12,13]. However, Urriola et al [9] reported that the GE concentration in corn distillers dried grains with solubles was underestimated when the GE was calculated using the prediction equation developed by Ewan [10]. To our knowledge, studies evaluating the accuracy of the previous prediction equations have been rather scarce. Therefore, the objectives of the present work were to evaluate the accuracy of prediction equations previously developed for estimating GE in feed ingredients and to develop the novel equations. Additionally, the novel equations were validated using the data that were not used for the model development.

## MATERIALS AND METHODS

### Dataset

A total of 2,279 ingredient samples were used to evaluate the previously published equations for predicting GE concentration in feed ingredients [10,11] and to develop novel prediction equations. The ingredient samples consisted of barley (n = 58), corn (n = 319), corn distillers dried grains with solubles (n = 13), corn gluten feed (n = 583), copra expellers (n = 156), copra meal (n = 234), cottonseed meal (n = 12), palm kernel expellers (n = 504), rapeseed meal (n = 114), soybean meal (n = 138), wheat (n = 70), and wheat bran (n = 78; Table 1). In the dataset, only 2,259 samples were available for validating the equation reported in Sauvant et al [11] due to the absence of data for crude fiber (CF) concentrations in 20 samples.

To validate the prediction equations developed in the present work and the previous studies [10,11], the GE and nutrient concentrations in feed ingredients from the swine NRC [13] were also utilized. Among 122 ingredients from the NRC [13], 38 ingredients were estimated for GE, 9 ingredients had no GE data reported, and 13 ingredients did not have ash concentration data. The remaining 62 ingredients were used for the validation of the equation by Ewan [10] and novel equations. Only 46 ingredients were used for validating the equation reported by Sauvant et al [11] as other 16 ingredients had no CF data.

### Chemical analyses

The ingredient samples were finely ground for chemical analyses (<1 mm). The concentrations of dry matter (DM; method 930.15), ash (method 942.05), crude protein (CP; method 990.03), ether extract (EE; method 920.39), and CF (method 962.09) were determined according to the AOAC [14]. The GE concentrations in the ingredient samples were analyzed using the adiabatic bomb calorimeter (Parr 1261; Parr Instruments Co., Moline, IL, USA).

### Statistical analyses

The accuracy of the previously published equations [10,11] was assessed as described by Choi et al [15]. The regression analyses were performed with measured minus predicted GE in the feed ingredients as a dependent variable and the predicted values minus the mean predicted value as an independent variable using the REG procedure of SAS (SAS Inst. Inc., Cary, NC, USA). In the linear regression, the intercept and the slope represent a mean bias and a linear bias, respectively. Novel equations for estimating GE concentrations in feed ingredients were developed using the REG procedure of SAS based on the data from 2,279 samples in which nutrient concentrations were regarded as independent variables. The accuracy of the equations developed in the present work and the previous equations was assessed using data of 62 ingredients from the NRC [13] as described above. The statistical significance and tendency were determined as p<0.05 and 0.05≤p<0.10, respectively.

## RESULTS

**Figure 1:**
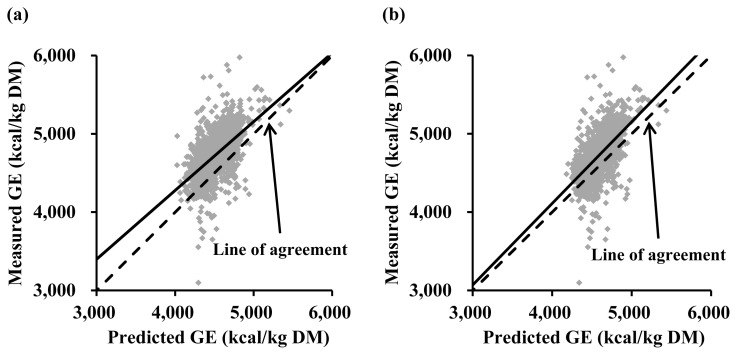
Evaluation of equations reported by Ewan [10] and Sauvant et al [11] for estimating gross energy (GE) concentrations (kcal/kg dry matter; DM) in feed ingredients. A regression analysis was performed for measured GE minus predicted GE on predicted GE minus average of predicted GE. (a) For the equation of Ewan [10], the intercept (218; standard error = 4 and p<0.001) and the slope (–0.121; standard error = 0.025 and p<0.001) were different from 0 based on 2,279 ingredient samples. (b) For the equation of Sauvant et al [11], the intercept (135; standard error = 4 and p<0.001) was greater than 0 but the slope (0.041; standard error = 0.028 and p = 0.143) was not different from 0 based on 2,259 ingredient samples.

The evaluation study based on the data from 2,279 ingredient samples indicated that the equation reported by Ewan [10] had both mean bias (intercept = 218; standard error = 4 and p<0.001) and linear bias (slope = −0.121; standard error = 0.025 and p<0.001; Figure 1a). The equation of Sauvant et al [11] also had the mean bias (intercept = 135; standard error = 4 and p<0.001; Figure 1b).

Based on the data from 2,279 ingredient samples, 3 prediction models for estimating GE concentration were developed using CP, EE, and ash as independent variables (Table 2). The best-fitting model was: GE, kcal/kg DM = 4,341+11×CP+ 54×EE–24×ash, with R^2^ = 0.372 and p<0.001 (Eq. 3), where the nutrient components are in % DM.

**Figure 2:**
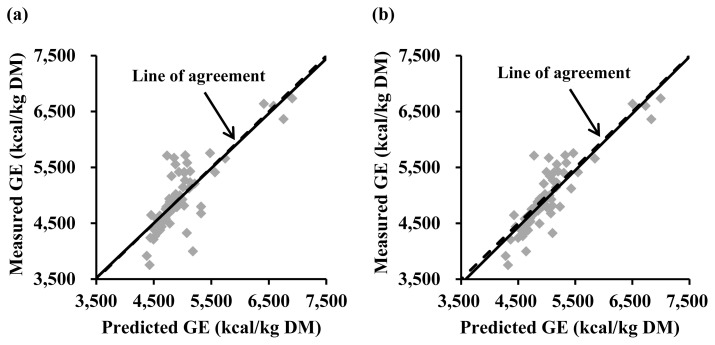
Validation of equations developed in the present study for estimating gross energy (GE) concentrations (kcal/kg dry matter; DM) in feed ingredients using 62 ingredients in the NRC [13]. A regression analysis was performed for measured GE minus predicted GE on predicted GE minus average of predicted GE. (a) For the Eq. 2, the intercept (–7; standard error = 47 and p = 0.884) and the slope (–0.018; standard error = 0.089 and p = 0.840) were not different from 0. (b) For the Eq. 3, the intercept (–56; standard error = 38 and p = 0.140) and the slope (0.014; standard error = 0.068 and p = 0.834) were not different from 0.

**Figure 3:**
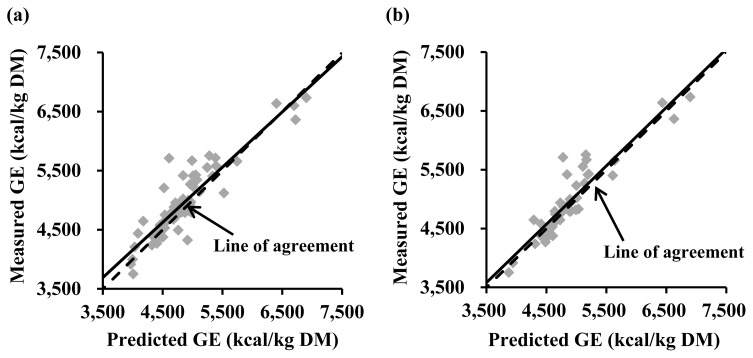
Evaluation of equations reported by Ewan [10] and Sauvant et al [11] for estimating gross energy (GE) concentrations (kcal/kg dry matter; DM) in feed ingredients. A regression analysis was performed for measured GE minus predicted GE on predicted GE minus average of predicted GE. (a) For the equation of Ewan [10], the intercept (101; standard error = 35 and p = 0.005) was greater than 0 but the slope (–0.066; standard error = 0.057 and p = 0.251) was not different from 0 based on 62 ingredients in the NRC [13]. (b) For the equation of Sauvant et al [11], the intercept (71; standard error = 36 and p = 0.056) tended to be greater than 0 but the slope (–0.007; standard error = 0.063 and p = 0.907) was not different from 0 based on 46 ingredients in the NRC [13].

A validation study based on the 62 ingredients from the swine NRC [13] indicated that the Eq. 2 did not have a mean bias (intercept = −7; standard error = 47 and p = 0.884) or a linear bias (slope = −0.018; standard error = 0.089 and p = 0.840) as presented in Figure 2a. The Eq. 3 also did not have any bias for intercept (−56; standard error = 38 and p = 0.140) or slope (0.014; standard error = 0.068 and p = 0.834; Figure 2b). However, the equation of Ewan [10] showed a mean bias (intercept = 101; standard error = 35 and p = 0.005; Figure 3a) and the equation reported by Sauvant et al [11] also had a mean bias tendency (intercept = 71; standard error = 36 and p = 0.056; Figure 3b).

## DISCUSSION

The DE, ME, and NE systems represent energy concentrations available to animals, and thus, these systems have been employed to formulate swine diets that meet energy requirement estimates of animals [3]. An accurate determination of DE, ME, or NE of a feed ingredient requires animal experiments that are time-consuming, laborious, and expensive. Alternatively, prediction models for estimating biologically available energy values of ingredients and diets for pigs using nutrient concentrations as independent variables are available [1,4,6,16,17]. It should be noted that the inclusion of GE concentration as an independent variable in the models for estimating the DE and ME improves the precision of the equations represented by increased determination coefficients [1,4,6]. In addition, the inclusion of ME as an independent variable in the equation for estimating NE increases the determination coefficient [8,18]. Therefore, an accurate GE value is essential for accurately estimating the DE, ME, or NE of a feed ingredient.

The underestimation of GE of feed ingredients observed in the present evaluation of the previous equations [10,11] is well supported by Urriola et al [9] who reported that the predicted GE in corn distillers dried grains with solubles using the equation of Ewan [10] was approximately 188 kcal/kg DM less than the measured GE. A linear bias observed in the evaluation of the equation by Ewan [10] indicates that the equation underestimates GE more for low-GE ingredients. The inaccuracy of the equation by Ewan [10] may be partially due to the limited number of ingredients (n = 33) and diet (n = 53) used for developing the equation. Nevertheless, the high determination coefficient (R^2^ = 0.98) of the equation of Ewan [10] may be attributed to the inclusion of some ingredients containing extremely high GE concentrations such as fats and oils.

The reason for the mean bias observed in the evaluation of the equation of Sauvant et al [11] remains unclear but is potentially associated with the differences in the characteristics of the ingredients. Although Sauvant et al [11] employed a large number of ingredient samples (n>2,000) for developing the equation, the ingredients included roughages as well as grains and oilseed meals whereas the ingredients used in the present evaluation study were mostly concentrate feeds (Table 1). In addition, the ingredients in the NRC [13] also are concentrate feed ingredients.

The 2,279 feed ingredients used for developing novel equations in the present study primarily consisted of grains, oilseed meals, and cereal by-products which are commonly used for non-ruminants. No bias observed in the validation of the novel equations using data from the swine NRC [13] suggests that GE concentrations in feed ingredients for non-ruminants can be more accurately estimated using the novel equations compared with the previous equations.

In the novel equations, only CP, EE, and ash concentrations were used as independent variables for estimating GE concentration. Although fiber, starch, or sugar as additional independent variables can potentially improve the accuracy of prediction of GE [11,19], the concentrations of these nutrients are often unavailable. As in the present work, Ewan [10] also employed only CP, EE, and ash concentrations as independent variables for estimating GE concentration.

## CONCLUSION

A previous equation for estimating gross energy concentrations in feed ingredients for non-ruminants underestimated the energy value determined using the adiabatic bomb calorimeter. In the present work, novel equations were developed for gross energy based on the chemical compositions of 2,279 ingredient samples for non-ruminants and the equations were fairly accurate based on the validation study.

## Figures and Tables

**Table 1:** Chemical composition of ingredients (dry matter basis)^1)^

Item	n	Mean	SD	Min.	Max.	CV
Moisture (%)	2,279	10.5	2.39	1.77	16.4	22.7
Gross energy (kcal/kg)	2,279	4,706	254	3,099	5,976	5.39
Crude protein (%)	2,279	21.6	10.7	7.24	54.6	49.8
Ether extract (%)	2,279	4.77	2.95	0.35	22.2	61.8
Crude fiber (%)	2,259	11.1	5.33	1.59	32.0	48.2
Ash (%)	2,279	5.60	2.53	0.70	14.3	45.2

SD, standard deviation; CV, coefficient of variation.

1) A total of 2,279 ingredient samples used in the present study consisted of barley (n = 58), corn (n = 319), corn distillers dried grains with solubles (n = 13), corn gluten feed (n = 583), copra expellers (n = 156), copra meal (n = 234), cottonseed meal (n = 12), palm kernel expellers (n = 504), rapeseed meal (n = 114), soybean meal (n = 138), wheat (n = 70), and wheat bran (n = 78).

**Table 2:** Prediction equation for gross energy concentration in feed ingredients (n = 2,279)^1)^

Item	Regression coefficient parameter				Statistical parameter		
	
	Intercept	CP	EE	Ash	RMSE	R^2^	p-value
Eq. 1	4,494	-	44	-	218	0.264	<0.001
Standard error	9	-	1.55	-	-	-	-
p-value	<0.001	-	<0.001	-	-	-	-
Eq. 2	4,299	7	53	-	206	0.342	<0.001
Standard error	14	0.43	1.57	-	-	-	-
p-value	<0.001	<0.001	<0.001	-	-	-	-
Eq. 3	4,341	11	54	–24	201	0.372	<0.001
Standard error	15	0.56	1.53	2.28	-	-	-
p-value	<0.001	<0.001	<0.001	<0.001	-	-	-

CP, crude protein; EE, ether extract; RMSE, root mean square error.

1) Gross energy concentration is expressed as kcal/kg dry matter and nutrient compositions are expressed as % dry matter.
